# Composite Repair on Zirconia: Influence of Different Sandblasting Pretreatments and Various Universal Adhesives on Shear Bond Strength

**DOI:** 10.3290/j.jad.c_1988

**Published:** 2025-04-16

**Authors:** Malin Janson, Vanessa Bassier, Anja Liebermann, Christoph Matthias Schoppmeier, Maria Di Gregorio-Schininà

**Affiliations:** a Malin Janson Dentist, University of Cologne, Faculty of Medicine and University Hospital Cologne, Department of Prosthetic Dentistry, Cologne, Germany. Conceptualization, methodology, validation, writing – original draft preparation, review and editing, supervision, project administration.; b Vanessa Bassier Dentist, Department of Prosthetic Dentistry, University of Cologne, Faculty of Medicine and University Hospital Cologne, Cologne, Germany. Validation, writing – original draft preparation, review and editing.; c Anja Liebermann Professor, dentist, Head of Department, Department of Prosthetic Dentistry, University of Cologne, Faculty of Medicine and University Hospital Cologne, Cologne, Germany. Conceptualization, supervision.; d Christoph Matthias Schoppmeier* Dentist, senior physician, Faculty of Medicine and University Hospital Cologne, Polyclinic for Operative Dentistry and Periodontology, University of Cologne, Cologne, Germany. Methodology, validation, writing – original draft preparation, review and editing.; e Maria Di Gregorio-Schininà* Dentist, senior physician, Department of Prosthetic Dentistry, University of Cologne, Faculty of Medicine and University Hospital Cologne, Cologne, Germany. Validation, writing – original draft preparation, review and editing, project administration. *Joint last authors

**Keywords:** repair, shear bond strength, universal adhesives, zirconia

## Abstract

**Purpose:**

This *in-vitro* study evaluated the effect of universal adhesives and sandblasting with 50 μm and 110 μm aluminum oxide particles (Al_2_O_3_) on the shear bond strength (SBS) between composite and zirconia in repair applications across different aging intervals.

**Materials and Methods:**

1296 zirconia specimens (Katana Zirconia HT) were randomized into three main groups: (A) sandblasting with 50 μm Al_2_O_3_, (B) sandblasting with 110 μm Al_2_O_3_, and (C) control. Each group was further divided into six subgroups: OPB (Optibond Universal), PBA (Prime&Bond Active), IBU (iBond Universal), CUBQ (Clearfil Universal Bond Quick), MBP (Monobond Plus), and SBUP (Scotchbond Universal Plus). Composite (Clearfil Majesty ES-2 Universal) was applied, and SBS (MPa) measured at baseline (24-h storage) at 30 and 90 days, and after 7 days + 5000 thermocycles (5–55°C). Failure modes were assessed at 40 × magnification. Analysis used a generalized linear model (GLM) with Bonferroni adjustment (α < 0.05).

**Results:**

Sandblasting significantly increased SBS compared to controls, with Group B showing the highest durability after thermocycling, with values decreasing over aging periods. In Groups A and B IBU (21.43 ± 2.7 MPa; 25.60 ± 5.78 MPa), SBUP (19.26 ± 3.2 MPa; 23.62 ± 4.4 MPa), and CUBQ (19.92 ± 2.8 MPa; 22.75 ± 4.34 MPa) achieved the highest SBS, with adhesive failures being predominant and cohesive failures mainly in high-SBS subgroups.

**Conclusion:**

Pretreatment with Al_2_O_3_ significantly enhances composite-zirconia bond strength, with larger grit sizes more effective. MDP-containing adhesives are recommended for reliable zirconia repairs.

Zirconia-based materials are widely used in restorative dentistry due to their excellent mechanical properties, such as high compressive strength, chemical stability, excellent biocompatibility, and a high modulus of elasticity.^2,18^ The introduction of digital CAD/CAM workflows has simplified the application process, allowing fast, straightforward digital design and manufacture of monolithic veneers and all-ceramics.^34^ In spite of the technological and scientific advances, zirconia restorations are not without their drawbacks. The most common complications are fractures and chipping. Particularly for small fractures, clinicians are challenged to decide whether to attempt a composite-adhesive repair or replace the entire restoration. ^29^ The latter inevitably leads to further irreversible loss of tooth structure due to re-preparation, so repair attempts are gaining importance due to their minimally invasive treatment approach and cost-effectiveness.^21^ Adhesion between composite and zirconia is challenging due to the inert zirconia surface, which makes it difficult to create microretention. This limits the use of composites for intraoral repairs due to disadvantages such as low bonding, durability, wear resistance, and poor esthetics.^6^ Acid-etching methods with hydrofluoric acid (HF) are considered the gold standard for glass-ceramic restorations. However, they are less effective on zirconia restorations due to their highly crystalline structure, inherent chemical stability, low surface energy, and lack of a glass phase. Various surface pretreatment methods are available for an effective micromechanical bond between composite and zirconia. These methods include surface abrasion with diamond burs, sandblasting with alumina particles, tribochemical silica coating or silicatization, and the use of CO_2_, Er:YAG, and Nd:YAG lasers.^13^ Sandblasting with different grit sizes is an effective, time-saving, and cost-efficient method to enhance adhesion by increasing surface roughness. This enlarges the bonding area and promotes micromechanical anchoring of the composite to zirconia, leading to an increased availability of hydroxyl groups for the reaction with MDP.^19,35^

Studies have shown that mechanical pretreatment alone is not sufficient for a durable bond.^19^ The lack of silica in zirconia can also make the use of conventional silane for bonding zirconia less effective. Therefore, researchers are increasingly focusing on the use of various universal adhesives in addition to established mechanical pretreatment methods.^30^ The chemical interaction between universal adhesives and zirconia is based on their functional adhesive monomers. These include 10-Methacryloyloxydecyldihydrogenphosphate (10-MDP), 3-Methacryloxypropyltrimethoxysilane (3-MPTS), 4-methacryloyloxyethyl trimellitate anhydride (4-META), or glycerol phosphate dimethacrylate (GPDM), which mainly consist of phosphate and/or carboxylate monomers. These monomers have the advantage of forming chemical bonds with zirconia, metals, and tooth structures by creating insoluble calcium salts, which ensure a durable and firm connection. Initially developed for adhesive and restorative applications on tooth hard tissues, MDP has become a key element in dental adhesive technology due to its broad applicability and ability to form stable salt bonds with the calcium ions of hydroxyapatite. MDP can be used not only on enamel and dentin but also as a bonding primer for various materials, including zirconia, silica-based ceramics, precious metals, non-precious metals, and composite resins. This combination eliminates the need for a separate silane and bonding agent, optimizing the clinical bonding process. Although initial results are promising, few studies have investigated the efficacy of different bonding agents specifically for zirconia, nor is there a universally accepted standard for pretreating monolithic zirconia restorations.^21,22^

Therefore, the objective of this study was to assess the efficacy of different surface pretreamtents (Al_2_O_3_ sandblasting with different grit sizes) in combination of various universal adhesives affected shear bond strength (SBS) between composite and zirconia in repairs after thermocyclic aging.

The study tested two null hypotheses:

The adhesion of composite and zirconia is not affected by the pretreatment method using sandblasting with different grit sizes of alumina particles.
The bond strengths between composite and zirconia are not influenced by the type of universal adhesive used.

## MATERIALS AND METHODS

The sample size was determined using G*Power software (Version 3.1.9.6, Franz Faul, University of Kiel, Germany). To achieve a statistical power of 80% with an effect size (Cohen’s d) of 0.32 and a significance level of 5% (α = 0.05), a total of 1236 samples was required, assuming an equal allocation (1:1 ratio) between the groups. To ensure better comparability and a more robust sample size, the final study included 1296 samples. A total of 1296 rectangular plates (10 x 10 x 2 mm) made of zirconia (Katana Zirconia HT, Kuraray Noritake, Okayama, Japan) were cut using a water-cooled milling machine (Ceramill Motion 2, Amann Girrbach, Koblach, Austria) and subsequently sintered according to the manufacturer’s instructions (Ceramill Therm 3, Amann Girrbach, Koblach, Austria).

The sample surfaces were polished under constant water cooling and at a steady pressure of 3 bar, using a progression from P600 up to P1200 grit silicon carbide foils (SiC Foil, Struers, Ballerup, Denmark), to achieve a standardized surface, subsequently subjected to ultrasonic cleaning in 96% isopropyl alcohol for 3 min, followed by steam cleaning for 10 s.^15^ Final thickness of each plate was determined using a digital caliper (Alpha Tools, Franklin, USA).

**Fig 1:**
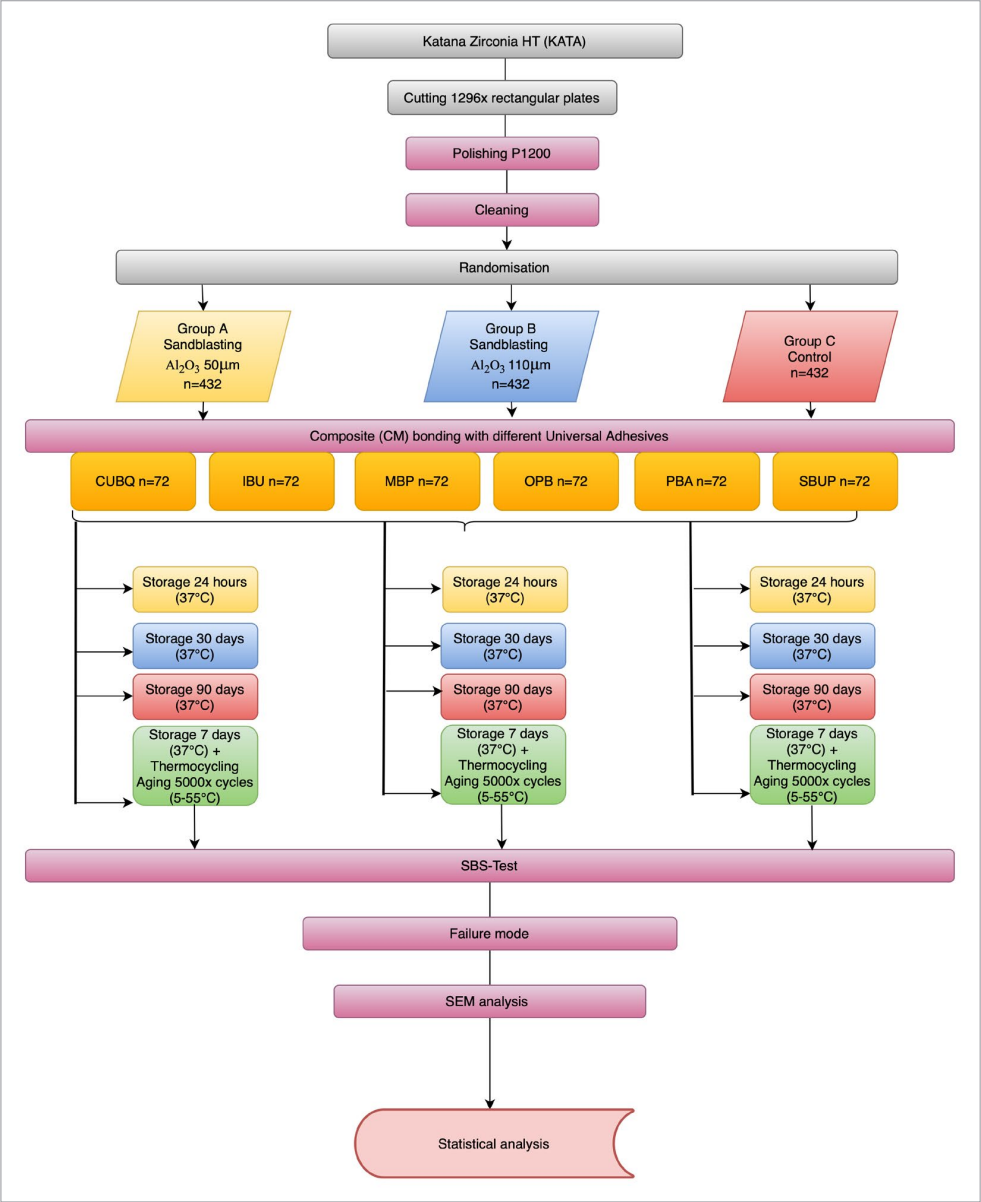
Study flowchart.

Details regarding the experimental groups and the methodologies employed for treatment are detailed within Figure 1. Further information on the brand names, manufacturers, batch numbers, the chemical composition and the application of the materials utilized in this study can be found in Table 1.

**Table 1:** Brand names, manufacturers, batch numbers, chemical composition and applications of materials used

Brand/Manufacturer	Abbreviation	Batch number	Chemical composition	Application
			Universal adhesives	
Katana Zirconia HT/Kuraray Noritake Dental, Okayama, Japan	KATA	EKQQH	Zirconium dioxide, ZrO_2_ (80.0–95.0%), yttrium oxide, Y_2_O_3_ (3.0–15.0%), titanium dioxide (0–10%), pigments.	–
Composite Clearfil Majesty ES-2 Universal Kuraray Noritake Dental Inc, Okayama, Japan	CM	430020	Silanated barium glass filler, pre-polymerized organic filler (0.0–40.0), Bisphenol A diglycidylmethacrylate, Hydrophobic aromatic dimethacrylate, Silanated colloidal silica/Hydrophobic aliphatic dimethacrylate, dl-Camphorquin	Applied and light cured in 2 x 1 mm increments
Korox 50, Bego GmbH, Bremen, Deutschland/ Dento-Prep; Ronvig, Daugaard, Dänemark	Group A		50 μm Al_2_O_3_-Particles	Pressure of 1 bar/0.1 MPa for 10 s at a distance of 10 mm
Korox 110, Bego GmbH, Bremen, Deutschland/ Cobra Aluoxyd, Renfert, Hilzingen, Germany	Group B		110 μm Al_2_O_3-_Particles	Pressure of 1 bar/0.1 MPa for 10 s at a distance of 10 mm
Calibra Silane + Prime&Bond active/Dentsply Sirona, Bensheim, Germany	PBA	2306000061 00124259	Phosphoric acid-modified acrylic resin, multifunctional acrylate, bifunctional acrylate, acid acrylate, isopropanol, water, initiators, stabilisators, acetone, ethyl alcohol, organosilane	Applied for 30 s, rinsed for 20 s, air dried Applied for 20 s, air dried for 5 s, light cured for 10 s
Clearfil Ceramic Primer Plus + Clearfil Universal Bond Quick/Kuraray Noritake Dental, Okayama, Japan	CUBQ	230091 220393	3-Trimethoxysilylpropylmethacrylat 10-Methacryloyloxydecyl-Dihydrogenphosphat, Ethanol MDP, Bis-GMA, HEMA, hydrophilic aliphatic dimethacrylate, colloidal silica, silane coupling agent, dl-camphor quinone, ethanol, water, Sodium fluoride	Applied Ceramic Primer, evaporated for 20 s, air dried Applied and massaged in for 10 s, air dried for 5 s, light cured for 10 s
Monobond Plus/Ivoclar Vivadent, Schaan, Liechtenstein	MBP	Z051GB	Phosphoric acid methacrylate, silane, methacrylate, sulfide methacrylate, alcohol	Applied for 60 s, air dried
Scotchbond Universal Plus/3M Deutschland GmbH Neuss, Germany	SBUP	9813336	10-MDP, HEMA, dimethacrylate polymers, Vitrebond copolymer, filler, ethanol, water, initiators, silane,	Applied for 20 s, air dried for 5 s, light cured for 10 s
iBond Ceramic Primer + iBond Universal/Kulzer, Hanau, Germany	IBU	N010125 MO10056	isopropanol, aceton Trimethyl-4,13-dioxo-3,14-dioxa-5,12-diaza-hexadecan-1,16-diylbismethacrylat, aceton, 4-Methacryloxyethyltrimellitanhydrid	Applied, evaporated for 20 s, air dried Applied, massaged in for 20 s, air dried, light cured for 10 s
Optibond Universal/Kerr Corporation, CA, USA	OPU	9426174	Aceton, 2-Hydroxyethylmethacrylat, glyercindimetacrylat, ethanol, glycerinphosphatdimethacrylat	Applied, massaged in for 20 s, air dried for 5 s, light cured for 10 s
Abbreviations: MDP, 10-Methacryloyloxydecyl-Dihydrogenphosphat; Bis-GMA, Bisphenol-A diglycidylmethacrylate; HEMA 2-Hydroxyethylmethacrylat

The specimens were randomly assigned to one of three distinct pretreatment categories (n = 432), each containing six subgroups (universal adhesives): (A) sandblasting with 50 μm aluminum oxide particles under 0.2 MPa pressure for 10 s, (B) sandblasting using 110 μm aluminum oxide particles under 0.2 MPa pressure for 10 s and (C) a control group that underwent no mechanical pretreatment on the ceramic surface. The six different universal adhesives (n = 72) were applied to the surface according to the manufacturer’s instructions using a microbrush by the same operator (VB) throughout the entire study.

Subsequently, composite was affixed onto the bonded sample surfaces. Samples were placed in a silicone mold to center a standardized Teflon mold (5 × 2 mm) on them. Composite (Clearfil Majesty ES-2 Universal, Kuraray Noritake, Okayama, Japan) was applied in two 1 mm increments and cured for 20 s each at 1 mm distance using an LED light curing unit (Bluephase Style, Ivoclar Vivadent, Ellwangen, Germany) with 1200 mW/cm^2^ intensity. After Teflon mold removal, samples underwent an additional 20-s polymerization.

All specimens were subjected to four distinct aging and measurement intervals: baseline (after 24 h of water storage at 37°C), 30 days of water storage, 90 days of water storage, and 7 days of water storage followed by thermocycling (5000 cycles, 5–55°C, 30-s dwell time, 5-s transfer time (RC 20 CS Lauda, Lauda-Königshofen, Germany).^10, 36^

SBS values, in MPa, were obtained using a universal testing machine (zwickiLine Z0.5 TN, Zwick Roell, Ulm, Germany) at a crosshead speed of 0.5 mm/min. Calculation of these values was performed by dividing the fracture load, measured in Newtons (N), by the bonded area in square millimeters (19,63 mm^2^ bonded area of each specimen). During testing, the bonding surface was aligned parallel to the loading mechanism, and shear force was applied at the composite-zirconia boundary by a knife-edge indenter, positioned as closely as possible to the interface to accurately gauge the bond strength.

Failure mode was assessed using a digital microscope (VHX-5000, Keyence Corp., Osaka, Japan) at 40 × magnification. This evaluation was carried out by two independent observers (CS, VB).

Failures were categorized into three distinct types:

Adhesive failure: This refers to the separation at the adhesive-zirconia interface.
Cohesive failure: This involves a breakdown within the composite material itself.
Mixed failure: This represents a combination of both adhesive and cohesive failures, occurring within the composite material.

For scanning electron microscopy (SEM) analysis, a zirconia sample from each group was selected, providing insights into the failure mechanisms at the interface. These samples were dried and coated with gold-palladium using a sputter coater (Q150T Plus, Quorum, Est Sussex, England) to enhance electron conductivity. SEM images were captured with a Prisma E SEM (Thermo Fisher Scientific, Waltham, MA, USA) at magnifications ranging from 100× to 10,000× (1536 × 1024 px) to observe surface details.

Data analysis was performed using IBM SPSS Version 29 (IBM, Armonk, NY, USA). The normality of data distribution was assessed using the Shapiro–Wilk test. To examine the effects of pretreatment measures (Control, 50 µm, 110 µm) and different aging conditions (24-h water storage, 30-day water storage, 90-day water storage, and 7-day water storage with 5000 thermocycling cycles) on the bond strength of universal adhesives, a generalized linear model (GLM) was applied. Due to the non-normally distributed and positive nature of the bond strength values, a gamma distribution with a logarithmic link function was chosen to ensure an appropriate model fit. Post-hoc tests with Bonferroni correction were conducted to identify significant differences between levels of pretreatment, and aging conditions with a significance level set at α <0.05.

## RESULTS

The Shapiro–Wilk test indicated significant deviations from normality (P < 0.01), and Levene’s test showed heterogeneity of variances (P < 0.001). Given these violations and positive bond strength values, a GLM with a gamma distribution and logarithmic link function was applied (SPSS Version 28). A significant interaction effect between pretreatments and adhesives was observed (P < 0.01).

Significant main effects were found for each primary factor: Abrasion (F(2) = 1696.833, P < 0.001, η² = 0.735), Aging (F(3) = 261.513, P < 0.001, η² = 0.391), and Adhesive (F(5) = 1324.402, P < 0.001, η² = 0.844). Interactions were significant between Abrasion and Aging (F(6) = 4.547, P < 0.001, η² = 0.022), Abrasion and Adhesive (F(10) = 55.353, P < 0.001, η² = 0.311), and Aging and Adhesive (F(15) = 7.552, P < 0.001, η² = 0.085). A three-way interaction was also significant between Abrasion, Aging, and Adhesive (F(30) = 1.718, P = 0.010, η^2^ = 0.040).

**Fig 2:**
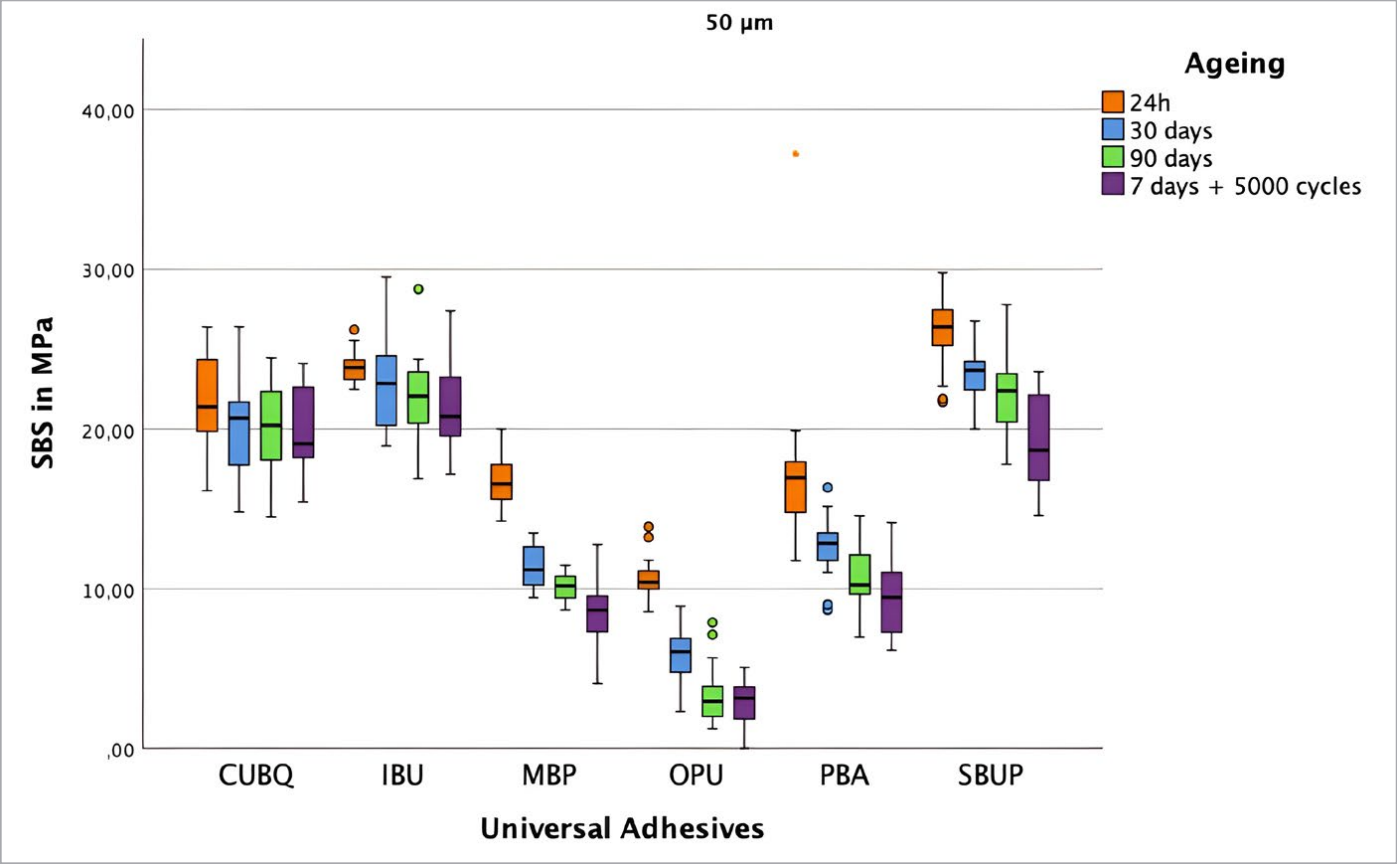
Shear bond strength values of the composite-ceramic interface using various universal adhesives with surface pretreatment group A (50 μm Al_2_O_3_), measured at 24 h (orange), 30 days (blue), 90 days (green), and after 7 days of storage plus 5000 thermocycling cycles (violet). Boxes represent the interquartile range, the central line indicates the median, and the whiskers show the full range of values. Outliers are marked with numbers and stars.

**Fig 3:**
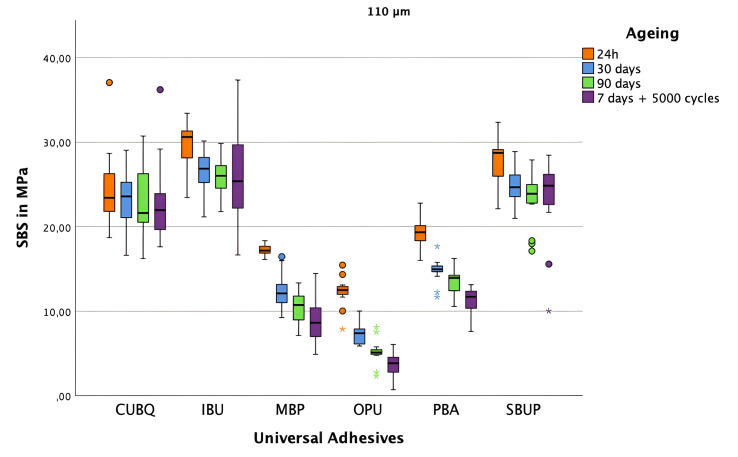
Shear bond strength values of the composite-ceramic interface using various universal adhesives with surface pretreatment group B (110 μm Al_2_O_3_), measured at 24 h (orange), 30 days (blue), 90 days (green), and after 7 days of storage plus 5000 thermocycling cycles (violet). Boxes represent the interquartile range, the central line indicates the median, and the whiskers show the full range of values. Outliers are marked with numbers and stars.

**Fig 4:**
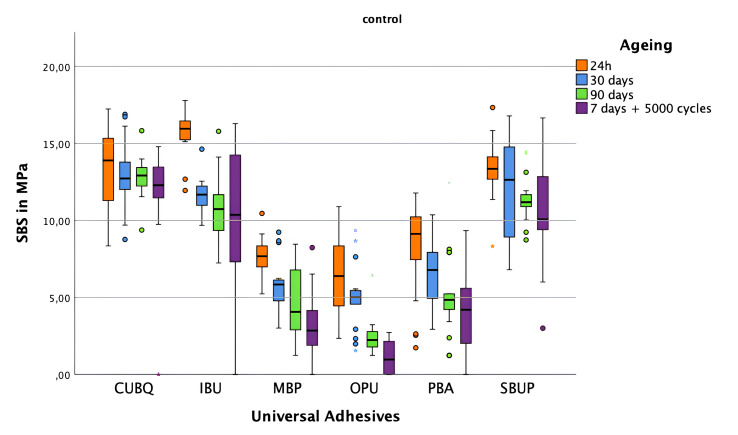
Shear bond strength values of the composite-ceramic interface using various universal adhesives with control group C (no surface pretreatment), measured at 24 h (orange), 30 days (blue), 90 days (green), and after 7 days of storage plus 5000 thermocycling cycles (violet). Boxes represent the interquartile range, the central line indicates the median, and the whiskers show the full range of values. Outliers are marked with numbers and stars.

Figures 2–4 present mean SBS values with standard deviations for each group. Groups treated with air abrasion achieved significantly higher SBS values than controls. SBS values decreased progressively over time, with significant reductions observed after both prolonged water storage (30 to 90 days) and thermocycling across all groups. At baseline (24-h water storage), the highest SBS in the control group was observed for IBU (15.64 ± 1.40 MPa), with SBUP leading in the 50 μm air abrasion group (26.04 ± 2.19 MPa) and IBU in the 110 μm group (29.66 ± 3.00 MPa) (Fig 4).

Pairwise comparisons confirmed significant differences across groups and aging stages. Notably, after thermocycling, IBU + 110 μm air abrasion showed the highest SBS (25.60 ± 5.78 MPa), followed by SBUP (23.62 ± 4.4 MPa) and CUBQ (22.75 ± 4.34 MPa) (Fig 3). The lowest SBS across f categories and aging conditions was observed for OPB after thermocycling (3.64 ± 1.36 MPa) (Fig 3). Detailed significance levels are provided in Table S1 of the supplementary data.

**Fig 5:**
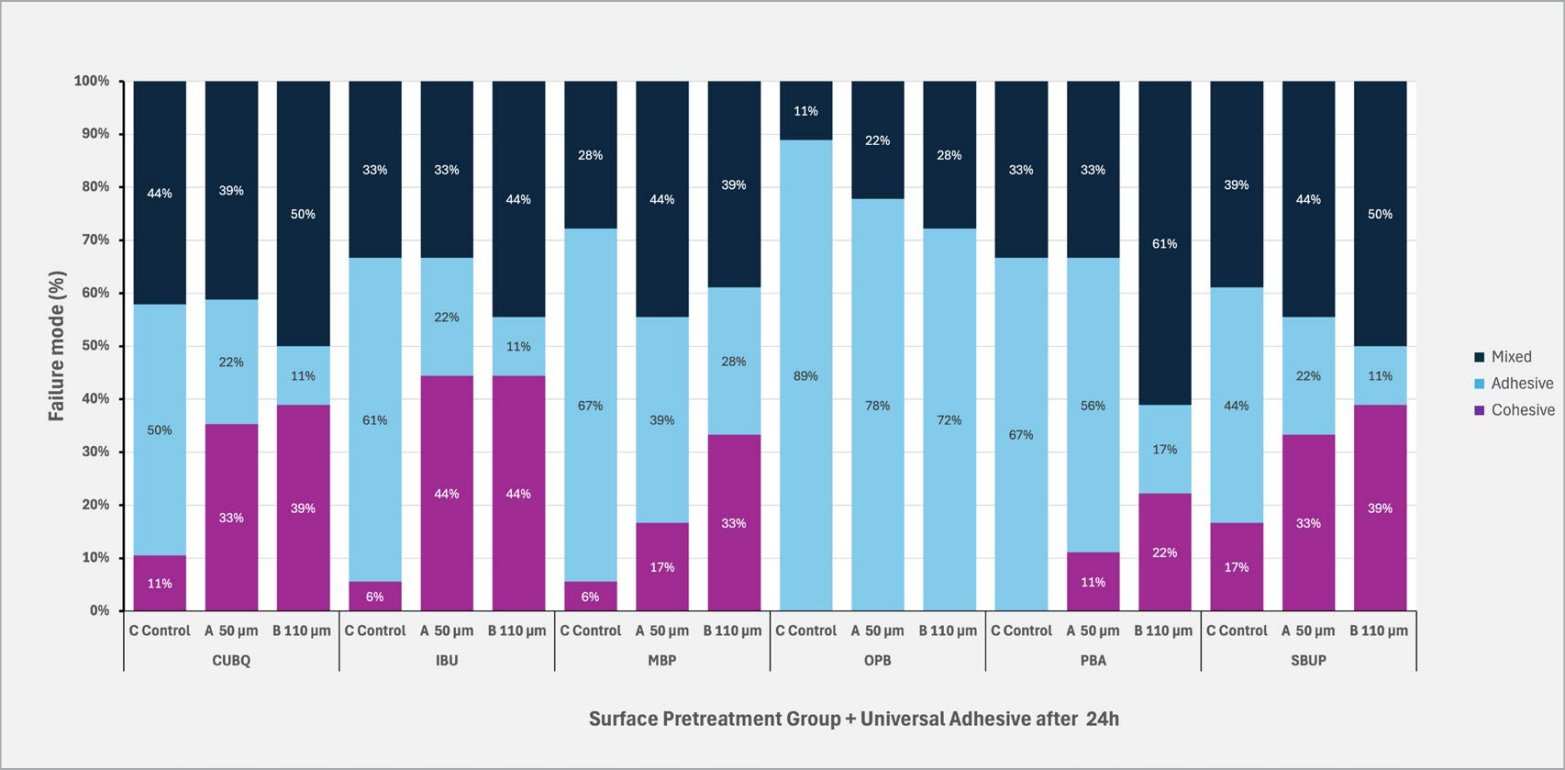
Failure mode distribution at baseline after 24 h for the three surface pretreatment groups and six universal adhesives.

**Fig 6:**
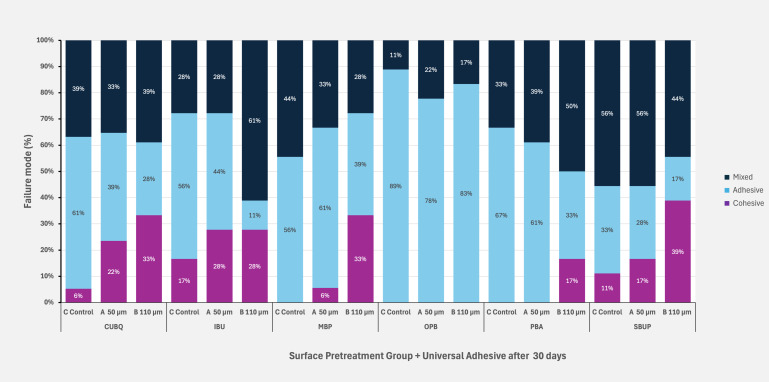
Failure mode distribution after 30 days water storage for the three surface pretreatment groups and six universal adhesives.

**Fig 7:**
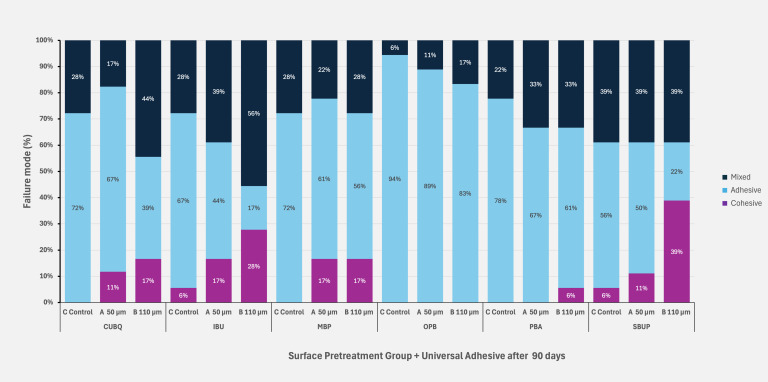
Failure mode distribution after 90 days water storage for the three surface pretreatment groups and six universal adhesives.

**Fig 8:**
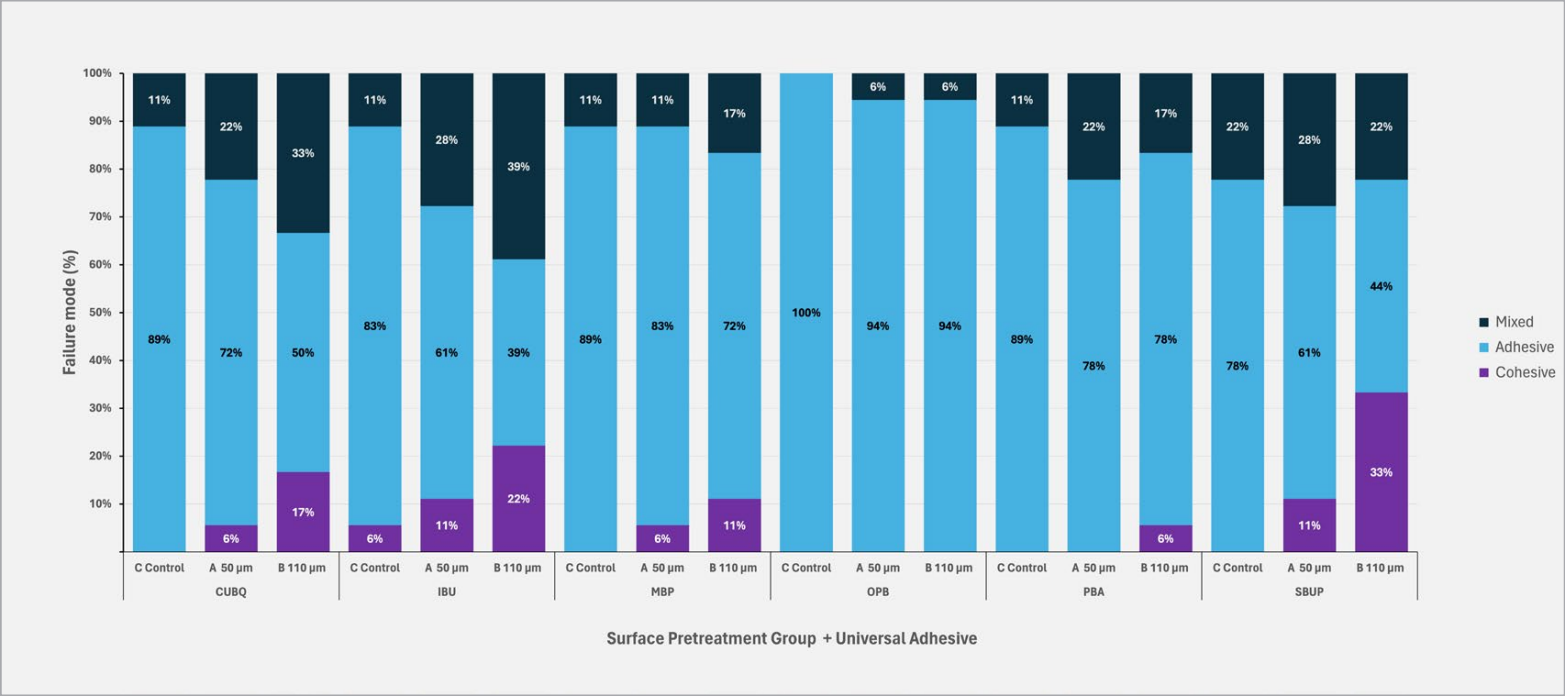
Failure mode distribution after 7 days water storage and 5000 × cycles for the three surface pretreatment groups and six universal adhesives.

Figures 5 to 8 illustrate the distribution of failure modes across all aging protocols, demonstrating a shift in failure patterns with increasing aging. Initially, baseline values exhibited a higher proportion of cohesive and mixed failures, particularly in groups with the highest SBS values, such as IBU, CUBQ, and SBUP. However, as aging progressed – through water storage to thermocycling – a marked increase in adhesive failures was observed, ranging from 62.83% to 88%.

**Fig 9:**
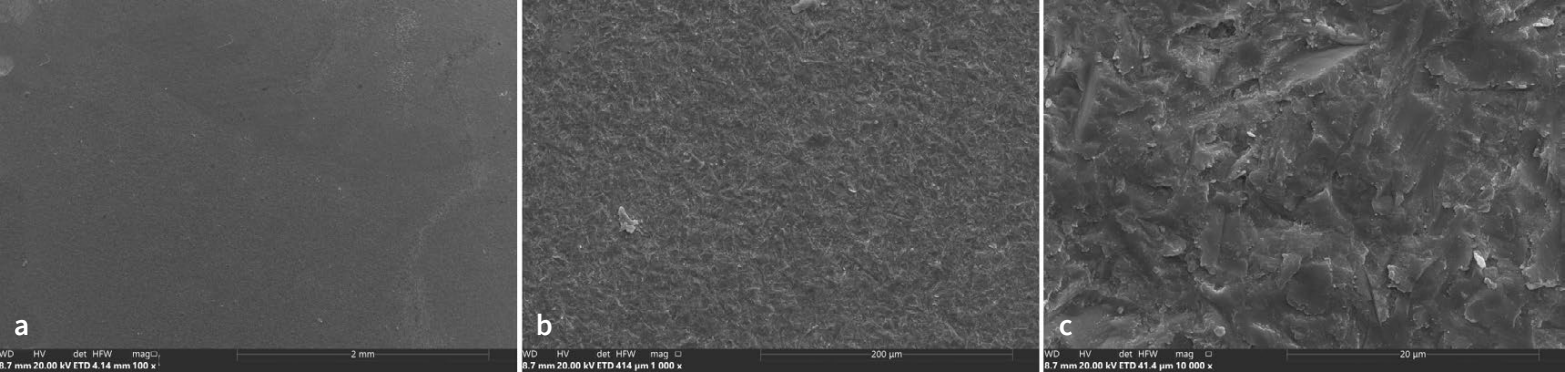

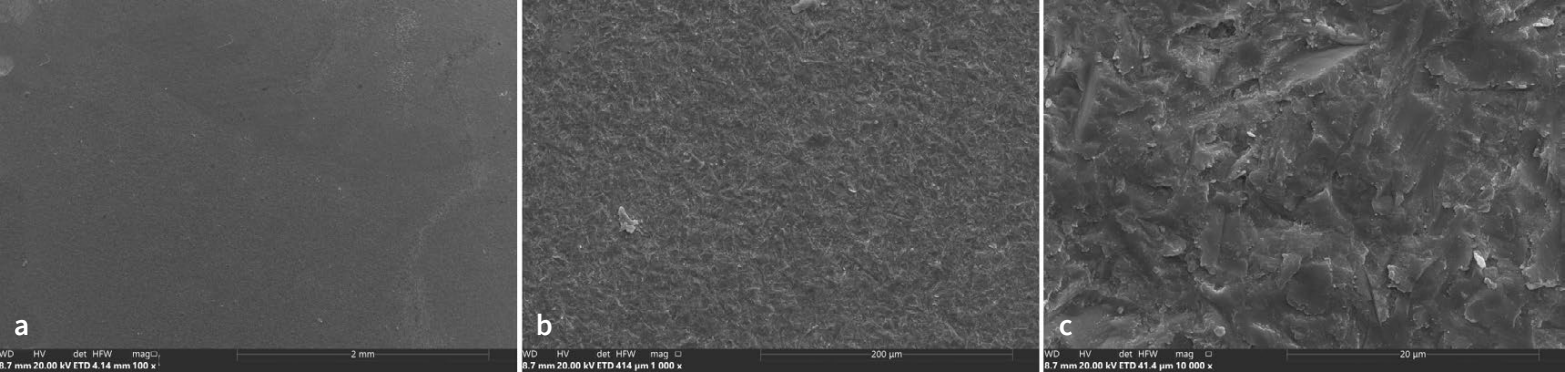
Micrograph of an adhesive failure specimen of OPB in the air-abraded 110ym group, indicating a KATA zirconia surface free of resin. The image is shown at three different magnifications: a, b, and c.

**Fig 11:**
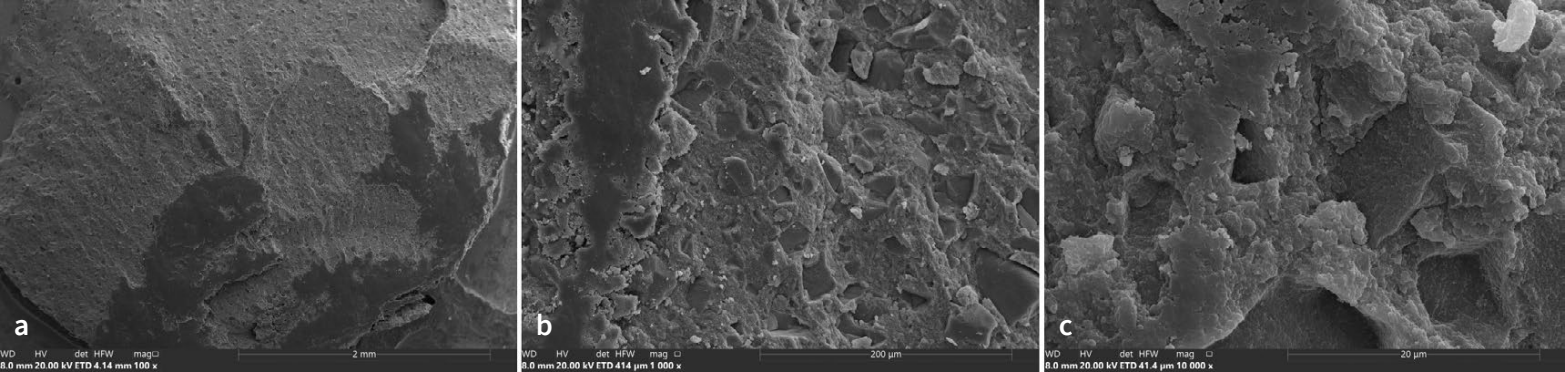
Micrograph of cohesive failure specimen of IBU in the air-abraded 110 ym group. The image shows composite on the surface of the KATA zirconium at three different magnifications: a, b, and c.

Mixed failures showed a notable increase in Groups A and B (19.5%/22.3%) after thermocycling, with Group B also exhibiting the highest incidence of cohesive failures (14.3%) compared to Groups A and C. This trend highlights the impact of aging on bond integrity, with adhesive failures becoming dominant under prolonged thermomechanical stress. SEM images of fractured composite-adhesive surfaces, highlighting these failure patterns, are shown in Figures 9 to 11.

## DISCUSSION

This study evaluated the impact of surface pretreatment using sandblasting with various grit sizes and different universal adhesives on the shear bond strength between composite and zirconia. The obtained results led to the rejection of both null hypotheses. The size of aluminum oxide particles and the type of universal adhesive used had an impact on adhesion. Therefore, it is evident that a combined mechanical and chemical pretreatment significantly affects the bond strength between composite and zirconia after thermocyclic aging.

For zirconia repairs, sandblasting with aluminum oxide particles alongside silica-coated Al_2_O_3_ is an established method for the surface pretreatment of zirconia.^24,38^ Systematic reviews emphasize the necessity of mechanochemical surface treatments involving particles of varying sizes in combination with functional monomers such as 10-MDP or 4-MET for reliable adhesion.^8,16^ However, the comparative effects of universal adhesives and particle sizes require further investigation.

Previous studies have indicated that the degree of surface roughness depends on various factors such as air pressure, particle size, and shape.^22,23,25^ with a particle size of 110 μm leading to increased surface roughness, while a size of 250 μm poses a risk of damaging the zirconia and should thus be avoided.^17,37^

The current study confirms that 50 μm and 110 μm aluminum oxide particles, applied at 0.2 MPa, significantly enhance bond strength compared to untreated surfaces, consistent with prior recommendations.^14^ Notably, Zicari et al reported that grit-blasting with 30 μm silica-coated Al_2_O_3_ improves bonding to veneering ceramics, potentially reducing chipping risks.^38^ Additionally, bond strength was significantly lower with 50 μm Al_2_O_3_ blasting compared to 110 μm Al_2_O_3_ blasting, suggesting a positive influence of particle size on the adhesive bond.

In the current study, aging reduced bond strength across all groups, yet samples treated with larger particle sizes retained notably higher bond strengths. This suggests that sandblasting with 110 µm particles provides a robust initial bond that withstands degradation more effectively. This finding aligns with clinical benchmarks, where bond strengths between 10 and 20 MPa are often considered sufficient for long-term durability. The preservation of acceptable bond levels after aging highlights the potential for reliable clinical performance with larger particle sizes in air abrasion protocols.

Our results are consistent with the study by Yang et al, which also highlighted reduced bond strength and increased spontaneous debonding in samples without particle blasting.^36^

The adhesion of composite materials to etched enamel is considered a critical benchmark, with bond strengths in the range of 15–30 MPa deemed adequate.^22^ According to ISO:10.477, the acceptable minimum SBS at the interface between resin-based materials and the substrate is 5 MPa. Conversely, the minimum acceptable bond strengths for ceramics and cementing agents are between 10 to 13 MPa,^1^ while other studies suggest 20 MPa as a clinically acceptable guideline.^23^ In terms of composite adhesion to zirconia, previous studies have shown bond strengths ranging from 16 to 50 MPa when blasted with 110 μm Al_2_O_3_ particles.^4^ Studies utilizing universal adhesives reported bond strengths varying between 5.95–29.35 MPa depending on the adhesive used, and in the current study between 2.76–25.6 MPa.^31^ The bond strengths of the control group without sandblasting were on average lower than after sandblasting, highlighting the effectiveness of combining sandblasting with the application of universal adhesives. This agrees with previous studies, as the bond strength with the sole application of universal adhesives without micromechanical modification did not constitute a sufficient bond.^16,23^

The decline in bond strength across all samples stored in water over 30 and 90 days and after thermocycling indicates that aging generally weakens the adhesive bond, independent of particle size. This trend is consistent with findings by Le et al, which noted that while sandblasting significantly increases bond strength, extended aging, including up to 150 days in water storage with 37,500 thermocycles, had little impact on sandblasted surfaces.^14^

In addition to mechanical pretreatment, to optimize adhesion strength, a chemical bond with MDP-based monomers, such as 10-MDP or 4-MET, in the form of a primer or universal adhesive, is recommended for stable and durable adhesion to zirconia.^11,22,26,33^ These specialized phosphate monomer-based primers enhance the chemical bond to zirconia through the interaction of the 10-MDP monomer with the zirconia surface. The chemical adhesion between composites and the hydroxyl groups is promoted via strong ionic and hydrogen bonds, and covalent bonds between oxygen, phosphorus, and zirconia on the zirconia surface.^7,19,23^ 10-MDP notably affects the adhesion to zirconia^19^ and ensures high bond strengths before and after thermocycling, regardless of the 10-MDP concentrations.^22^ The results of the current study suggest that universal adhesives exhibit varying behaviors and not all universal adhesives demonstrate definitive reliability in the adhesive bond. Therefore, the choice of universal adhesives impacts the long-term success of zirconia repairs with composites. The MDP-containing adhesives CUBQ, SBU, and IBU showed higher bond strengths compared to other studied universal adhesives. The values for CUBQ are comparable with study results that led to SBS of 26.34 MPa.^9^ Liebermann et al demonstrated that the bond strengths of CUBQ to lithium disilicate ceramics averaged at 21.5 MPa.^15^ In contrast, adhesives such as MBP and PBA achieved less optimal, yet acceptable adhesion values. These adhesives rely on phosphoric acid methacrylate as the primary bonding agent. While this agent does facilitate bonding through a mechanism involving the chelation with metal oxides present on the zirconia surface, it typically does not form as strong or as durable bonds as those formed by 10-MDP. The lower bond strengths with PBA could also be explained by the absence of 10-MDP and the presence of acetone. Some adhesives contain additional fillers or additives that can influence the viscosity, curing properties, and the stability of the bond. Particularly noteworthy in this study is the low adhesive bond in the OPB groups, which, according to ISO:10.477, did not lead to a sufficient composite-zirconia connection. The inferior performance observed in OPB can be attributed to the absence of 10-MDP. Although OPB contains GPDM, a glycerol phosphate dimethacrylate that theoretically promotes adhesion between composite materials and zirconia, the bonding efficacy of GPDM alone does not match that of the more effective 10-MDP. This result underscores the significance of selecting appropriate monomers for enhancing adhesive formulations to improve the bonding strength and longevity on zirconia substrates. Further investigations into the concentration of GPDM used as an equivalent to 10-MDP could provide insights into its lesser adhesion efficiency. According to Yoshihara, 10-MDP remains the most effective component for adhesion.^5^ Overall, the comparative analysis of these adhesive systems highlights the critical role of monomer composition in achieving high-quality adhesion. The inclusion of 10-MDP in adhesive formulations is particularly advantageous for applications involving zirconia ceramics,^20^ emphasizing the need for careful selection and optimization of monomers in dental adhesives to enhance performance and outcomes in clinical settings.

The analysis of failure modes aligned with the results from the SBS tests.

No visible damage to the zirconia surface was observed in any of the groups. In the control group without sandblasting, adhesive failure modes predominated, corroborating the findings of previous studies. Overall, adhesive failures were the most prevalent failure type in this study. Pretreatment with Al_2_O_3_ particles resulted in a higher incidence of mixed failures compared to the control group without air abrasion. Mixed failures are associated with higher bond strengths compared to purely adhesive failure rates.^3^ Generally, a cohesive loss between ceramic and composite is preferable, as it correlates with higher adhesion values. The subgroups with the highest SBS values (CUBQ, IBU, SBUP) exhibited a higher proportion of cohesive fractures, indicating a strong adhesive bond.

To evaluate the adhesive strength of zirconia to bonding agents, various testing methods were employed, including shear, tensile, push-out adhesion tests, and the Brazil nut method. This *in-vitro* study focused on the SBS test, which has been established as a reliable method for evaluating the adhesion performance between composites and ceramics, including zirconia.^12,22,28^

Finally, analysis of failure modes revealed that adhesive failures were most prevalent in groups undergoing long-term aging, regardless of surface treatment. The untreated control groups showed a higher percentage of adhesive failures compared to sandblasted groups, aligning with previous findings. This failure mode distribution underscores the necessity of both mechanical and chemical pretreatment for achieving durable adhesive bonds, particularly when long-term stability under aging conditions is critical for clinical success.

The assessment of adhesive strength was conducted under simulated long-term conditions to ensure the integrity of the adhesive interface in a moist oral environment and to prevent debonding. Despite existing ISO guidelines (ISO 4049:2009, ISO 29022:2013), the lack of a uniform protocol for artificial aging complicates the comparability of results.^27^ In this study, an aging protocol with multiple intervals was employed (water storage at 37°C for 24 h, 30 days, 90 days, and thermocycling) to gain a comprehensive understanding of the adhesive performance over time. A thermocyclic aging of at least 5000 cycles is considered an adequate method to simulate the long-term stability of composite-zirconia bonds.^22^ This approach provides insights into the stability of composite-zirconia bonds under thermal and hydrolytic stress, with 5000 cycles and seven days of water storage at 37°C representing approximately six months of clinical function, as per Gale and Darvell.^10^

The study results revealed a progressive decline in bond strength across all aging intervals, with significant reductions observed following thermocycling. This trend aligns with previous findings by Tsuo et al., indicating that adhesive bonds weaken with increased aging duration regardless of particle size for surface sandblasting. Larger particles (110 μm) resulted in increased surface roughness, which correlates with higher initial bond strength; however, the durability of these bonds diminished progressively with aging.^32^ Notably, groups treated with 110 μm Al_2_O_3_ particles maintained the highest SBS values even after thermocycling, suggesting that surface preparation using larger grit sizes could enhance durability in the early stages of restoration.

Although the use of a 5000-cycle thermocycling protocol represents a meaningful benchmark, it represents only a fraction of the expected clinical lifespan of dental restorations. Furthermore, limitations of *in-vitro* studies, such as the lack of simulated oral conditions like variable pH levels, salivary flow, temperature fluctuations, and masticatory forces, restrict the direct applicability of these results to clinical practice. No artificial aging of the zirconia substrates was conducted prior to testing, which previous studies suggest may lead to lower adhesive values over time. The comparability of different studies is further limited due to the diversity of materials used – including ceramics, composites, and universal adhesives – and the variety of applied techniques.

The variability of the adhesive bond depends on the used universal adhesive and the type of ceramic, underscoring the importance of material composition. Additional aspects such as marginal fit, color stability, and the role of specific components in the universal adhesives, particularly the monomer MDP in combination with other ingredients, require further scientific investigation to optimize the durability and applicability of these adhesion processes in clinical practice.

## CONCLUSIONS

A combined mechanical and chemical pretreatment significantly impacts the bond strength between composite materials and zirconia, though SBS decreases progressively with aging. Specifically, surface pretreatment of zirconia using aluminum oxides with increasing grit sizes (50 μm and 110 μm) enhances the bond strength of repair composites. Additionally, universal adhesives containing MDP exhibit superior SBS values and are recommended for use with sandblasting in intraoral repairs of zirconia restorations.

### Clinical Relevance

For optimal intraoral repairs of zirconia restorations, universal adhesives containing MDP are recommended due to their superior SBS values when used with sandblasting using aluminum oxide grit sizes of 50 μm and 110 μm.

**Fig 10:** Micrograph of mixed failure specimen of PBA in the air-abraded 110 ym group. The image shows the residual composite and resin on the surface of the KATA zirconia at three different magnifications: a, b, and c.

**Table 2:** Shear bond strength values (Mean + Standard Deviation in MPa) of universal adhesives used according to surface pretreatments

Universal Adhesive	Surface Pretreatment Group
A Al2O3 50 μm	B Al2O3 110 μm	C Control
CUBQ	19.92 2.61^cde^	22.75 4.34^cde^	11.82 3.26^cdeAB^
IBU	21.37 2.54^cde^	25.60 5.78^cde^	10.01 4.82^cdeAB^
MBP	8.44 2.03^abdf^	8.91 2.35^abdf^	2.99 2.26^abfAB^
OPB	2.76 1.63^abcef^	3.64 1.36^abcef^	1.10 1.01^abf^
PBA	9.51 2.13^abdf^	11.31 1.47^abdf^	3.89 2.44^abfAB^
SBUP	19.26 2.97^cde^	23.62 4.46^cdeB^	10.46 3.25^cdeAB^
Total	13.54 7.39	15.97 9.15	6.71 5.15
Small letters indicate significant differences (p < 0.05) between the universal adhesives within each surface pretreatment group. Capital letters indicate significant differences (p < 0.05) between the surface pretreatments within the universal adhesives. Abbreviations: CUBQ, Clearfil Universal Bond Quick; IBU, iBond Universal; MBP, Monobond Plus; OPB, Optibond Universal; PBA, Prime & Bond active; SBUP, Scotchbond Universal Plus
